# Acute Respiratory Distress Syndrome in Patients with Lymphopenia: Results from the National Inpatient Sample (2017–2021)

**DOI:** 10.3390/jcm14145148

**Published:** 2025-07-20

**Authors:** Arnav Garyali, Trishna Parikh, Dhruv Kumar, Ishan Gupta, Adishwar Rao, Akriti Agrawal, Sabiha Armin, Rishi Panjala, Rohan Patil, Nikhil Sriram, Sruthi Parthasarathy, Aarohi Parikh, Bindu Akkanti

**Affiliations:** 1 Baylor College of Medicine, One Baylor Plaza, Houston, TX 77030, USA; arnav.garyali@bcm.edu; 2 Department of Internal Medicine, Case Western Reserve University/University Hospitals of Cleveland, Lakeside Building, 3rd Floor Suite, 11100 Euclid Avenue, Cleveland, OH 44106, USA; 3 College of Natural Sciences, University of Texas at Austin, 2515 Speedway, Austin, TX 78712, USA; dk28392@utexas.edu; 4 Arizona College of Osteopathic Medicine, Midwestern University, Ocotillo Hall, 19555 59th Ave, Glendale, AZ 85308, USA; ishan.gupta@uth.tmc.edu; 5 Department of Internal Medicine, Robert Packer Hospital/Guthrie Clinic, 1 Guthrie Sq, Sayre, PA 18840, USA; adishwar.rao@gurthie.org (A.R.); akriti.agrawal@guthrie.org (A.A.); 6 Department of Internal Medicine, McGovern Medical School at The University of Texas Health Science Center at Houston, 6431 Fannin St, MSB 1.150, Houston, TX 77030, USA; sabiha.armin01@gmail.com; 7 Department of Psychological & Brain Sciences, Texas A&M University, Psychology Building, Building 0463, 515 Coke St, College Station, TX 77843, USA; rp1147@tamu.edu; 8 McGovern Medical School at The University of Texas Health Science Center at Houston, 6431 Fannin St, Houston, TX 77030, USA; rohan.r.patil@uth.tmc.edu; 9 Feinberg School of Medicine, Northwestern University, 420 E Superior St, Chicago, IL 60611, USA; nikhil.sriram@northwestern.edu; 10 Center for Advanced Heart Failure, The University of Texas Health Science Center at Houston, 6431 Fannin, MSB 1.150, Houston, TX 77030, USA; sruthi.parthasarathy@uth.tmc.edu (S.P.); bindu.h.akkanti@uth.tmc.edu (B.A.); 11 Department of Internal Medicine, HCA Houston Healthcare Kingwood/University of Houston College of Medicine, 22999 US 59 N, Kingwood, TX 77339, USA; parikhaarohi.md@gmail.com; 12 Division of Pulmonary, Critical Care and Sleep Medicine, Department of Internal Medicine, McGovern Medical School at The University of Texas Health Science Center at Houston, 6431 Fannin, MSB 1.150, Houston, TX 77030, USA

**Keywords:** acute respiratory distress syndrome, coronavirus disease 2019, lymphopenia, in-hospital mortality

## Abstract

**Background:** Lymphopenia has been associated with in-hospital, early, and late mortality. We aimed to elucidate differences in baseline characteristics in patients with lymphopenia with and without acute respiratory distress syndrome (ARDS) and determine predictors of in-hospital mortality in this patient population. **Methods:** Patients ≥ 18 years of age with lymphopenia were identified in the National Inpatient Sample (2017–2021) and stratified according to ARDS diagnosis. Predictors of in-hospital mortality were determined using multivariate analyses with a logistic regression model. **Results:** From 183,185 patients with lymphopenia, 10,420 (5.7%) had ARDS, of which 92.8% had coronavirus disease 2019. The patients with ARDS suffered from more in-hospital mortality (47% versus 6.7%, *p* < 0.001). ARDS increased the odds of in-hospital mortality by eight-fold (odds ratio [OR]: 7.91 [7.06–8.86], *p* < 0.001). Age ≥ 65 years (OR: 4.88 [3.98–5.99]), moderate/severe liver disease (OR: 2.53 [1.87–3.42]), and metastatic cancer (OR: 2.18 [1.68–2.82]) were among the strongest positive predictors of in-hospital mortality (all *p* < 0.001). **Conclusions:** Patients with lymphopenia who have ARDS have higher in-hospital mortality, likely due to the condition’s clinical course. Lymphopenia may be a marker of immune dysregulation and systemic involvement in ARDS.

## 1. Introduction

Immune dysregulation can occur among critically ill intensive care unit (ICU) patients, including higher neutrophil/lymphocyte ratios; reduced natural killer (NK) cells; and reduced T cells, particularly CD4+ T cells [1]. Lymphopenia, characterized by low lymphocyte counts with varying cutoffs, has been shown to have prevalences of 38% and 34% in all-cause hospitalizations and ICU admissions, respectively [2]. Although one study found no association between lymphopenia and infections [2], another large prospective study found 26% to 126% increases in the risks of different infections, including pneumonia, urinary tract infections, and diarrheal disease [3]. Additionally, lymphopenia, especially persistent lymphopenia, can predispose individuals to secondary infections [4] with subsequent worse outcomes, including longer lengths of stay and mortality [5]. Lymphopenia has been associated with higher mortality across multiple conditions [2,6,7,8,9].

Acute respiratory distress syndrome (ARDS) is a severe, life-threatening condition with several inciting etiologies such as pneumonia, pulmonary contusion, sepsis from a non-pulmonary source, and pancreatitis [10]. Pneumocyte and alveolar injuries prompt a cascade of events leading to an inflammatory response and vascular leakage, resulting in hypoxemia and the need for invasive mechanical ventilation [10,11]. Immune alterations have been studied in patients with ARDS. While one study found that lymphopenia was not a predictor of ARDS in severe acute respiratory syndrome (SARS) (not coronavirus disease 2019 [COVID-19]) [12], other studies have analyzed the prognostic value of immune cell count ratios in ARDS, including the lymphocyte/neutrophil ratio [13] and the neutrophil to lymphocyte and platelet ratio [14]. However, studies regarding clinical features of lymphopenia and ARDS are limited.

Given the pervasiveness of lymphopenia in hospitalized patients, the morbidity and mortality of patients with ARDS, and the paucity of research describing associations between lymphopenia and ARDS in terms of clinical characteristics, we aimed to (1) determine differences in baseline characteristics between those with and without ARDS in a cohort of patients with lymphopenia and (2) identify predictors of in-hospital mortality, including ARDS, in the entire cohort. We used the National Inpatient Sample (NIS), where lymphopenia was captured in patients with ARDS.

## 2. Methods

### 2.1. Dataset and Patient Population

The NIS is a publicly available all-payer inpatient database provided by the Agency for Healthcare Research and Quality through the Healthcare Cost and Utilization Project (HCUP) [15]. It has information on demographic, clinical, economic, and hospital characteristics.

**Figure 1:**
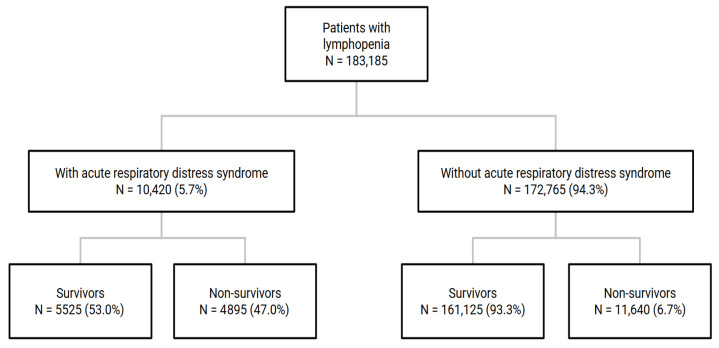
Survival of patients with lymphopenia according to acute respiratory distress syndrome (ARDS) diagnosis. Patients with lymphopenia were stratified according to whether they had coexisting acute respiratory distress syndrome. In-hospital mortality in each group is also shown.

We identified patients who were at least 18 years of age with a primary (main reason for hospitalization) or secondary diagnosis (coded during hospitalization) of lymphopenia using the International Classification of Diseases, Tenth Revision, Clinical Modification (ICD-10-CM) [16] code D72.810 during the years 2017 to 2021 (D72.810 reflects a clinical diagnosis of lymphopenia as documented by the treating provider). The patients were further stratified based on whether they had ARDS using the ICD-10-CM code J80 (Figure 1). Patients with missing data on in-hospital mortality were excluded. We conducted all analyses on weighted estimates, given the stratified sampling design of the NIS, using the DISCWT variable provided by the HCUP.

This study was conducted in accordance with the principles outlined by the Declaration of Helsinki (1975, revised in 2013). Institutional Review Board approval was waived, given that the NIS is de-identified and publicly available [17].

### 2.2. Data Collection and Outcome Measurements

Baseline demographic, economic, and healthcare utilization measures and hospital, and clinical characteristics were obtained. Several chronic comorbidities were evaluated, as was COVID-19, a common cause of ARDS. A Charlson Comorbidity Index (CCI) of ≥4 indicated multimorbidity. The primary outcome of interest was in-hospital mortality.

### 2.3. Statistical Analysis

We assessed the normality of the data using histograms, box plots, and QQ plots. The baseline characteristics of those with and without ARDS were compared using the Pearson chi-square test for categorical variables and the two-sample *t*-test for continuous variables. Categorical variables are presented as frequencies (percentages), while continuous variables are presented as means (standard deviations). Multivariate analysis with a logistic regression model adjusted for demographics and confounders was used to identify predictors of in-hospital mortality. The aforementioned variables of interest were assessed as predictors, except for total charges, total charges adjusted for yearly inflation, total cost, and calendar year, which were not assessed as predictors. ARDS was assessed as a predictor. Adjusted odds ratios (ORs) with corresponding 95% confidence intervals and *p*-values are presented. A *p*-value <0.05 was regarded as statistically significant. Stata 18 (StataCorp, College Station, TX, USA) was used to perform all analyses.

## 3. Results

### 3.1. Baseline Characteristics

Among 183,185 hospitalized patients with lymphopenia, 10,420 (5.7%) had coexisting ARDS (Table 1). The patients with ARDS were more likely to be male (63.5% versus 55.4%, *p* < 0.001) and younger (61.13 [14.71] years versus 61.97 [17.51] years, *p* < 0.001). Myocardial infarction (11.6% versus 9.1%, *p* < 0.001), uncomplicated diabetes (27.6% versus 17.2%, *p* < 0.001), and hemiplegia or paraplegia (2.3% versus 1.5%, *p* = 0.005) were more prevalent among the patients with ARDS. Severe acute respiratory syndrome coronavirus 2 (SARS-CoV-2)/COVID-19 was found in 92.8% and 45.5% of the patients with and without ARDS, respectively (*p* < 0.001). The patients with ARDS (47.0%) suffered more from in-hospital mortality than those without ARDS (6.7%) (*p* < 0.001).

### 3.2. Predictors of In-Hospital Mortality and Survival

ARDS was the strongest predictor of in-hospital mortality (OR: 7.91 [7.06–8.86], *p* < 0.001), followed by age ≥ 65 years compared to age 18 to less than 45 years (OR: 4.88 [3.98–5.99], *p* < 0.001) (Table 2). Moderate/severe liver disease (OR: 2.53 [1.87–3.42], *p* < 0.001) and metastatic cancer (OR: 2.18 [1.68–2.82], *p* < 0.001) were associated with in-hospital mortality. Acquired immunodeficiency syndrome (OR: 1.55 [0.97–2.49], *p* = 0.069) and COVID-19 (OR: 0.88 [0.74–1.04], *p* = 0.133) showed no significant associations. Determinants of survival included age less than 45 years; female sex (OR: 0.77 [0.70–0.83], *p* < 0.001); length of stay < 7 days; hospitals in the Midwest region compared to the Northeast region (OR: 0.85 [0.76–0.96], *p* = 0.009); and the absence of comorbidities such as moderate/severe liver disease, metastatic cancer, congestive heart failure, and ARDS.

## 4. Discussion

We found that among patients with lymphopenia, those with ARDS suffered more frequently from in-hospital mortality; ARDS served as a major predictor of in-hospital mortality. Despite its high prevalence among patients with ARDS, COVID-19 was not associated with in-hospital mortality. Moderate/severe liver disease and metastatic cancer joined ARDS in predicting in-hospital mortality in the entire cohort.

The existing literature describes associations between lymphopenia and adverse outcomes, particularly with sepsis/septic shock, where lymphopenia may be driven by decreased lymphocyte and lymphocyte precursor production in the bone marrow and thymus, migration of lymphocytes to sites of infection, or destruction via cell death or apoptosis [18]. Clinically, lymphopenia was bidirectionally associated with septic shock [2,6]. Among hospitalized patients with pneumococcal community-acquired pneumonia (CAP), those with an absolute lymphocyte count <500/mm^3^ were more likely to be admitted to the ICU and develop septic shock [9]. Patients with CAP, sepsis, and concomitant lymphopenia had longer hospital stays and higher in-hospital mortality and 30-day mortality [7].

Sepsis is often an antecedent to ARDS [10]. ARDS is characterized by an exudative phase with interstitial and alveolar edema, a restorative or proliferative phase, and a possible fibrotic stage; this underlying pathophysiology can lead to the need for mechanical ventilation if there is refractory hypoxemia. The high mortality rate and increased odds of in-hospital mortality in our study may be due to the inherent nature of ARDS itself, where the mortality rate can be as high as 45% in severe cases [10], or the antecedent condition, such as sepsis. Furthermore, baseline immunosuppression may be playing a role, as immunocompromised patients with ARDS have higher in-hospital mortality than immunocompetent patients despite comparable ARDS severity [19]. While we cannot determine the prognoses of patients with ARDS and lymphopenia compared to those with only ARDS, a question that warrants further study, we show that ARDS is a multisystem condition or one step in the process of multiorgan failure [11]. The immune dysregulation exemplified in this condition may be a prognostic indicator of weaning failure [20] and mortality [13,14].

Nearly all patients with ARDS had a coded diagnosis of COVID-19 in our study. The SARS-CoV-2 virus induces lymphopenia through the processes of cell destruction and suppression of lymphopoiesis [21], and changes in lymphocyte count and function and cytokine/chemokine release occur. Patients with severe COVID-19 displayed lower lymphocyte counts, a greater neutrophil/lymphocyte ratio, decreased T-lymphocyte counts, and signs of T-cell exhaustion [21,22]. Monocytic and granulocytic myeloid-derived suppressor cells (MDSCs) were inversely correlated with the T-cell count, suggesting a role in T-cell dysfunction [22]. Patients with COVID-19, severe pneumonia, or ARDS had elevated levels of interleukin (IL)-6 [22,23], IL-10 [22,23], and granulocyte colony-stimulating factor (G-CSF) [22,24,25]. In fact, one study focusing on the immunopathology of COVID-19 versus non-COVID-19 ARDS found that C-X-C motif chemokine ligand-10 (CXCL10), granulocyte–macrophage colony-stimulating factor (GM-CSF), and IL-10 were related to COVID-19 ARDS, while a second cluster that included IL-6 was related to the Sequential Organ Failure Assessment (SOFA) score [24].

The high prevalence of COVID-19 suggests this is the most likely cause of ARDS, although other etiologies and incidental COVID-19 infections are not excluded. Lymphopenia in the setting of COVID-19 is associated with worse outcomes. Lymphopenia was associated with severe COVID-19 [26] and an increased risk [27] or odds [28] of ARDS. Lower lymphocyte counts were noted among those who had severe COVID-19 [26], suffered from ARDS, required ICU care [26,29], or died [26,29]. Lymphopenia was associated with invasive mechanical ventilation; dialysis; and in-hospital mortality [29], including in-hospital mortality in immunosuppressed individuals [28]. Our study differs in that we found no association between COVID-19 and in-hospital mortality among patients with lymphopenia. This could be related to the severity of the infection. Additionally, if lymphopenia is appropriately coded in all COVID-19 patients, statistically significant associations may emerge. Together these findings highlight the intersection between COVID-19, lymphopenia, and ARDS, potentially identifying a group of patients who would benefit from interventions to restore immunological balance and reduce hyperinflammation [30] or to re-establish immune competency [30,31].

Moderate/severe liver disease and metastatic cancer significantly predicted in-hospital mortality, joining ARDS as two of the strongest predictors based on comorbidities. Liver dysfunction impairs proinflammatory cytokine clearance and synthesis of essential proteins such as clotting factors and acute-phase reactants, predisposing patients to coagulopathy and infections [32]. Critically ill patients with lymphopenia and liver cirrhosis exhibited significantly higher mortality rates compared to those without cirrhosis [33]. Similarly, patients with cancer and concomitant lymphopenia experienced worse outcomes [34], possibly due to increased vulnerability to infections, a decreased capacity to recover from critical illnesses [35], poor nutritional status, reduced functional reserve, and increased disease burden.

We used a nationally representative dataset to examine the associations between lymphopenia, ARDS, and in-hospital mortality. However, there are limitations to this study. An administrative database is subject to potential errors in coding, including undercoding of lymphopenia. We did not have clinical data (e.g., vital signs and organ failure assessments) to determine the baseline conditions (e.g., the SOFA scores) of the patients with ARDS. A threshold for lymphopenia could not be established, given the use of the NIS, which may have led to variability in clinical interpretation and underreporting of lymphopenia. Details regarding lymphopenia, such as the timing of onset, severity, and persistence, could not be determined. The patients may have had lymphopenia due to a pre-existing immunologic vulnerability (patients with a baseline immunosuppressed state were not excluded), as an acquired feature of a critical illness, or as a marker of disease severity. Although the etiology of ARDS was not obtained, it was likely secondary to COVID-19; thus, the findings may not be generalizable to those with non-COVID-19 ARDS. Further studies should divide patients with lymphopenia into those who have non-COVID-19 ARDS and those who have COVID-19 ARDS. Given the cross-sectional, discharge-level National Inpatient Sample (NIS) database, the absence of longitudinal follow-up data precludes survival curve estimation. There are likely unmeasured confounders due to the usage of administrative codes within this retrospective study. Given the administrative nature of the dataset, future prospective, granular data can be applied to potentially highlight more robust causal inference methods and stratification.

## 5. Conclusions

Patients with lymphopenia who develop ARDS suffer more from in-hospital mortality, with ARDS serving as an independent predictor of in-hospital mortality. While it is likely secondary to the inherent nature of ARDS, the immune dysregulation caused by inciting conditions, and possibly ARDS itself, cannot be understated, highlighting the systemic disease process. Lymphopenia may be a marker of this impaired immune response and could serve as a prognostic marker. Moderate/severe liver disease and metastatic cancer, both known to be associated with lymphopenia, worsened outcomes in patients with lymphopenia. Future studies should focus on the efficacy of therapies targeting the immune response to improve outcomes in patients with ARDS, developing cost-effective ARDS management techniques, and implementing interventions to optimize major and minor comorbidities.

## Figures and Tables

**Table 1:** Baseline characteristics of patients with lymphopenia with and without acute respiratory distress syndrome.

	ARDS	No ARDS	Total	*p*-Value
N	10,420 (5.7%)	172,765 (94.3%)	183,185 (100.0%)	
Sex				<0.001
Male	6620 (63.5%)	95,675 (55.4%)	102,295 (55.8%)	
Female	3800 (36.5%)	77,090 (44.6%)	80,890 (44.2%)	
Age in years at admission (y)	61.13 (14.71)	61.97 (17.51)	61.92 (17.36)	0.024
Age group				<0.001
18 to less than 45 years	1455 (14.0%)	30,335 (17.6%)	31,790 (17.4%)	
45 to less than 65 years	4445 (42.7%)	59,070 (34.2%)	63,515 (34.7%)	
≥65 years	4520 (43.4%)	83,360 (48.3%)	87,880 (48.0%)	
Race				<0.001
White	4755 (47.3%)	96,950 (57.6%)	101,705 (57.0%)	
Black	1555 (15.5%)	29,090 (17.3%)	30,645 (17.2%)	
Hispanic	2615 (26.0%)	28,430 (16.9%)	31,045 (17.4%)	
Asian or Pacific Islander	435 (4.3%)	5940 (3.5%)	6375 (3.6%)	
Native American	100 (1.0%)	1035 (0.6%)	1135 (0.6%)	
Other	600 (6.0%)	6990 (4.1%)	7590 (4.3%)	
Length of stay (days)	18.98 (13.66)	7.32 (8.07)	7.99 (8.91)	<0.001
Length of stay, grouped				<0.001
<7 days	1235 (11.9%)	107,280 (62.1%)	108,515 (59.2%)	
≥7 days	9185 (88.1%)	65,485 (37.9%)	74,670 (40.8%)	
Primary expected payer/insurance				<0.001
Medicare	4320 (41.5%)	85,620 (49.6%)	89,940 (49.1%)	
Medicaid	1820 (17.5%)	27,530 (16.0%)	29,350 (16.0%)	
Private insurance	3395 (32.6%)	46,350 (26.9%)	49,745 (27.2%)	
Self-pay	440 (4.2%)	6955 (4.0%)	7395 (4.0%)	
No charge	45 (0.4%)	590 (0.3%)	635 (0.3%)	
Other	390 (3.7%)	5545 (3.2%)	5935 (3.2%)	
Region of hospital				0.051
Northeast	2050 (19.7%)	41,510 (24.0%)	43,560 (23.8%)	
Midwest	3055 (29.3%)	47,585 (27.5%)	50,640 (27.6%)	
South	2875 (27.6%)	50,505 (29.2%)	53,380 (29.1%)	
West	2440 (23.4%)	33,165 (19.2%)	35,605 (19.4%)	
Bed size of hospital				0.845
Small	2265 (21.7%)	36,475 (21.1%)	38,740 (21.1%)	
Medium	2545 (24.4%)	43,920 (25.4%)	46,465 (25.4%)	
Large	5610 (53.8%)	92,370 (53.5%)	97,980 (53.5%)	
Location/teaching status of hospital				0.140
Rural	400 (3.8%)	9935 (5.8%)	10,335 (5.6%)	
Urban non-teaching	1505 (14.4%)	24,655 (14.3%)	26,160 (14.3%)	
Urban teaching	8515 (81.7%)	138,175 (80.0%)	146,690 (80.1%)	
Total charges (USD)	298,132.96 (312,065.67)	90,182.45 (138,502.68)	101,968.03 (161,010.78)	<0.001
Total charges (USD) adjusted for yearly inflation	305,667.25 (321,230.59)	93,061.34 (143,324.11)	105,110.72 (166,252.04)	<0.001
Total cost (USD)	71,275.78 (71,430.22)	20,814.30 (28,874.74)	23,674.19 (34,809.09)	<0.001
Calendar year				<0.001
2017	15 (0.1%)	6690 (3.9%)	6705 (3.7%)	
2018	45 (0.4%)	8095 (4.7%)	8140 (4.4%)	
2019	55 (0.5%)	8475 (4.9%)	8530 (4.7%)	
2020	5510 (52.9%)	86,360 (50.0%)	91,870 (50.2%)	
2021	4795 (46.0%)	63,145 (36.5%)	67,940 (37.1%)	
Comorbidities				
Myocardial infarction	1210 (11.6%)	15,705 (9.1%)	16,915 (9.2%)	<0.001
Congestive heart failure	1830 (17.6%)	35,210 (20.4%)	37,040 (20.2%)	0.004
Peripheral vascular disease	460 (4.4%)	10,835 (6.3%)	11,295 (6.2%)	<0.001
Cerebrovascular disease	455 (4.4%)	8305 (4.8%)	8760 (4.8%)	0.383
Dementia	510 (4.9%)	13,950 (8.1%)	14,460 (7.9%)	<0.001
Chronic obstructive pulmonary disease	2410 (23.1%)	43,710 (25.3%)	46,120 (25.2%)	0.035
Rheumatoid disease	300 (2.9%)	7525 (4.4%)	7825 (4.3%)	0.002
Peptic ulcer disease	130 (1.2%)	1990 (1.2%)	2120 (1.2%)	0.696
Mild liver disease	535 (5.1%)	11,270 (6.5%)	11,805 (6.4%)	0.016
Moderate/severe liver disease	90 (0.9%)	3105 (1.8%)	3195 (1.7%)	0.001
Uncomplicated diabetes	2875 (27.6%)	29,665 (17.2%)	32,540 (17.8%)	<0.001
Diabetes with chronic complications	1510 (14.5%)	25,145 (14.6%)	26,655 (14.6%)	0.940
Hemiplegia or paraplegia	240 (2.3%)	2590 (1.5%)	2830 (1.5%)	0.005
Renal disease	1830 (17.6%)	37,600 (21.8%)	39,430 (21.5%)	<0.001
Cancer (metastatic)	65 (0.6%)	6125 (3.5%)	6190 (3.4%)	<0.001
Cancer (other)	240 (2.3%)	9745 (5.6%)	9985 (5.5%)	<0.001
Acquired immunodeficiency syndrome	65 (0.6%)	2680 (1.6%)	2745 (1.5%)	<0.001
COVID-19	9670 (92.8%)	78,645 (45.5%)	88,315 (48.2%)	<0.001
Charlson Comorbidity Index				<0.001
<4	8500 (81.6%)	128,755 (74.5%)	137,255 (74.9%)	
≥4	1920 (18.4%)	44,010 (25.5%)	45,930 (25.1%)	
Died during hospitalization	4895 (47.0%)	11,640 (6.7%)	16,535 (9.0%)	<0.001

The baseline characteristics of the entire cohort of patients with lymphopenia, stratified by the presence of coexisting acute respiratory distress syndrome. ARDS: acute respiratory distress syndrome; COVID-19: coronavirus disease 2019.

**Table 2:** Predictors of in-hospital mortality in patients with lymphopenia.

Predictor	Odds Ratio (95% Confidence Interval)	*p*-Value
Female	0.77 (0.70–0.83)	<0.001
Age group		
18 to less than 45 years	reference	—
45 to less than 65 years	2.18 (1.81–2.63)	<0.001
≥65 years	4.88 (3.98–5.99)	<0.001
Race/Ethnicity		
White	reference	—
Black	1.22 (1.09–1.37)	0.001
Hispanic	1.22 (1.09–1.37)	0.001
Asian or Pacific Islander	0.90 (0.71–1.13)	0.344
Native American	1.41 (0.87–2.29)	0.161
Other	1.40 (1.16–1.70)	<0.001
Length of stay ≥ 7 days	1.96 (1.80–2.15)	<0.001
Primary expected payer		
Medicare	reference	—
Medicaid	1.04 (0.88–1.22)	0.655
Private insurance	0.90 (0.79–1.03)	0.109
Self-pay	1.27 (0.99–1.64)	0.063
No charge	1.20 (0.55–2.62)	0.652
Other	1.14 (0.90–1.45)	0.264
Region of hospital		
Northeast	reference	—
Midwest	0.85 (0.76–0.96)	0.009
South	0.90 (0.80–1.01)	0.075
West	1.00 (0.88–1.14)	0.986
Bed size of hospital		
Small	reference	—
Medium	1.19 (1.06–1.34)	0.004
Large	1.10 (0.99–1.22)	0.083
Location/teaching status of hospital		
Rural	reference	—
Urban non-teaching	1.28 (1.02–1.60)	0.034
Urban teaching	1.17 (0.95–1.44)	0.146
Acute respiratory distress syndrome	7.91 (7.06–8.86)	<0.001
Comorbidities		
Myocardial infarction	1.56 (1.38–1.76)	<0.001
Congestive heart failure	1.51 (1.36–1.68)	<0.001
Peripheral vascular disease	1.05 (0.90–1.24)	0.526
Cerebrovascular disease	1.27 (1.07–1.50)	0.007
Dementia	1.52 (1.34–1.72)	<0.001
Chronic obstructive pulmonary disease	1.11 (1.01–1.22)	0.029
Rheumatoid disease	0.93 (0.73–1.17)	0.529
Peptic ulcer disease	0.90 (0.60–1.35)	0.601
Mild liver disease	1.07 (0.89–1.28)	0.469
Moderate/severe liver disease	2.53 (1.87–3.42)	<0.001
Uncomplicated diabetes	1.15 (1.03–1.28)	0.01
Diabetes with chronic complications	1.29 (1.12–1.48)	<0.001
Hemiplegia or paraplegia	1.60 (1.19–2.14)	0.002
Renal disease	1.31 (1.14–1.49)	<0.001
Cancer (metastatic)	2.18 (1.68–2.82)	<0.001
Cancer (other)	1.21 (0.98–1.48)	0.072
Acquired immunodeficiency syndrome	1.55 (0.97–2.49)	0.069
COVID-19	0.88 (0.74–1.04)	0.133

Predictors of in-hospital mortality in patients with lymphopenia. Determined via multivariate analysis using a logistic regression model. COVID-19: coronavirus disease 2019.

## Data Availability

The datasets used to generate the results of this study are not available upon request due to mandates from the Healthcare Cost and Utilization Project.

## Abbreviations

The following abbreviations are used in this manuscript:

ARDS	acute respiratory distress syndrome
CAP	community-acquired pneumonia
CCI	Charlson Comorbidity Index
COVID-19	coronavirus disease 2019
CXCL	C-X-C motif chemokine ligand
G-CSF	granulocyte colony-stimulating factor
GM-CSF	granulocyte–macrophage colony-stimulating factor
HCUP	Healthcare Cost and Utilization Project
ICD-10-CM	International Classification of Diseases, Tenth Revision, Clinical Modification
ICU	intensive care unit
IL	interleukin
MDSC	myeloid-derived suppressor cell
NIS	National Inpatient Sample
NK	natural killer
OR	odds ratio
SARS	severe acute respiratory syndrome
SARS-CoV-2	severe acute respiratory syndrome coronavirus-2
SOFA	Sequential Organ Failure Assessment
